# Clinical, Histological, and Molecular Prognostic Factors in Childhood Medulloblastoma: Where Do We Stand?

**DOI:** 10.3390/diagnostics13111915

**Published:** 2023-05-30

**Authors:** Charikleia Ntenti, Konstantinos Lallas, Georgios Papazisis

**Affiliations:** 1 First Department of Pharmacology, School of Medicine, Aristotle University of Thessaloniki, 54621 Thessaloniki, Greece; 2 Department of Medical Oncology, School of Medicine, Aristotle University of Thessaloniki, 54621 Thessaloniki, Greece; 3 Clinical Research Unit, Special Unit for Biomedical Research and Education (BRESU), School of Medicine, Aristotle University of Thessaloniki, 54621 Thessaloniki, Greece

**Keywords:** medulloblastoma, tumor, histologic, pediatrics, micro-RNAs, non-coding RNAs, prognosis, molecular subgroup, stratification

## Abstract

Medulloblastomas, highly aggressive neoplasms of the central nervous system (CNS) that present significant heterogeneity in clinical presentation, disease course, and treatment outcomes, are common in childhood. Moreover, patients who survive may be diagnosed with subsequent malignancies during their life or could develop treatment-related medical conditions. Genetic and transcriptomic studies have classified MBs into four subgroups: wingless type (WNT), Sonic Hedgehog (SHH), Group 3, and Group 4, with distinct histological and molecular profiles. However, recent molecular findings resulted in the WHO updating their guidelines and stratifying medulloblastomas into further molecular subgroups, changing the clinical stratification and treatment management. In this review, we discuss most of the histological, clinical, and molecular prognostic factors, as well the feasibility of their application, for better characterization, prognostication, and treatment of medulloblastomas.

## 1. Introduction

Medulloblastomas (MBs) are highly aggressive neoplasms of the central nervous system (CNS) and are considered the most common malignant brain tumor in childhood [1,2]. First described by Cushing and Bailey in 1925, these tumors derive from discrete neuronal lineages based on molecularly defined subgroups, as shown by recent studies. For example, the cells of origin for the SHH subgroup are the granule-neuron progenitors, whereas for Groups 3 and 4, early rhombic lip is considered the common source of origin [3,4,5].

Epidemiologically, the estimated annual incidence of the tumor in the US is approximately 500 cases per year and the median age of diagnosis is 6–8 years; it is very rarely diagnosed in adults [6].

Since 1990, when the estimated event-free survival (EFS) of MBs was 20–50% [7], and with the application of newer therapeutic techniques, the median overall survival of all subtypes is estimated to be 70% [8,9,10]. The tumor presents a significant biological heterogeneity, as it has been observed that about 30% of patients will be diagnosed with metastatic disease at presentation, and patients who survive may be diagnosed with subsequent malignancies during their life or develop treatment-related neurocognitive, endocrinological, or development disorders, highlighting the need for risk stratification of patients with MB. The aim of risk stratification is the appropriate selection and management of patients who can really benefit from treatment or who need treatment intensification. Clinical and histological factors were the initial determinants of prognosis and the main parameters used for patient categorization into standard and high risk [11,12]. However, the development of new molecular techniques has revolutionized the prognostication of MBs, creating new molecular subgroups with distinct clinical features, response to treatment options, and prognosis. Around 2010, several researchers reported that medulloblastomas comprise at least four distinct molecular subgroups: wingless signaling activated (WNT), Sonic-hedgehog signaling activated (SHH), Group 3, and Group 4, largely based on transcriptome profiles and a few known genetic alterations [13,14,15]. Thereafter, for the first time in 2016, molecular subgroups of MBs were incorporated into the WHO’s MB stratification [16,17]. The WNT subgroup accounts for approximately 10% of all MBs, whereas the SHH subgroup is most common in infants and young adults, accounting for 25% of all MBs [15,18]. Group 3 and Group 4 constitute approximately 65% of medulloblastoma cases and are characterized by great heterogeneity in clinical phenotypes and survival rates [19,20]. The most recent edition of CNS tumor classification (CNS 5), from 2021, divided medulloblastomas into “molecularly” and “histologically” defined, denoting the diverse biology of the tumor [21].

However, although recent research has mainly focused on a thorough investigation of molecular mechanisms behind MB pathogenesis and their contribution to risk stratification of patients, clinicopathological characteristics are also important factors for both relapse and prognosis and have been involved in recently developed prognostic nomograms [22].

The main scope of our review is to summarize the evidence concerning clinical, pathological, and molecular factors affecting the prognosis of childhood medulloblastomas. We mainly focus on the interconnection between these factors and their role in risk stratification, aiming to prove that appropriate treatment selection in chosen patients is feasible.

## 2. Methodology

We searched the literature via PubMed, Scopus, and Cochrane for relevant studies, using specific keywords such as “medulloblastoma”, “prognosis”, and “prognostic factors” from inception to January 2023. Studies referring to clinicopathological and molecular factors associated with the prognosis of childhood medulloblastomas were included, whereas studies referring to adulthood or not referring to prognosis, as well as non-English papers, were excluded from the reviewing process. The primary outcome of our review was to investigate possible interactions between clinical, histologic, and molecular characteristics of MBs and the prognosis of the patients. In addition, the effect of the aforementioned factors on relapse occurrence and their association with post-relapse prognosis were set as secondary outcomes.

## 3. Results

### 3.1. Clinical and Histological Prognostic Factors

Before the establishment of molecular subgroups of MBs, patient risk stratification was mainly carried out based on their clinical characteristics, the histological features of the tumor, and the treatment approaches followed. Using the above factors as prognostic parameters, patients are categorized into two discrete subtypes: standard and high risk. Age < 3 years old, residual tumor > 1.5 cm^2^, and large-cell/anaplastic histology are considered high-risk features, whereas patients not fulfilling the above criteria are considered standard risk [9,12,23].

### 3.2. Age

One of the clinical characteristics used for risk stratification is the age of the patient. In general, younger age at diagnosis seem to have a worse prognosis compared to older children and is considered a high-risk feature [24]. More precisely, infants and children < 3 years showed the worst prognosis, and various studies have investigated survival rates of those patients [25]. Rutkowski et al. reported a hazard ratio (HR) of 4.02 (95% CI 1.28–12.96) for higher risk for relapse or death for children below 2 years of age compared to children 2–3 years old, whereas results from a Canadian study concluded that children older than 18 months had better survival rates compared to infants below that threshold [26,27]. A possible explanation could be the avoidance of craniospinal irradiation of the tumor in that age group due to the defects that radiotherapy cause in the developing brain. Despite that, a recently published meta-analysis and a retrospective study from Brazil did not reach a statistical significance for age as a poor prognostic factor [8,28,29]. On the other hand, MBs in adults demonstrate a lower mutational rate, are less aggressive, and are associated with better prognosis compared to younger patients [30].

### 3.3. Extent of Disease

Another significant factor that affects the prognosis of childhood MBs and is included in the risk-stratification system is the extent of disease. Chang‘s staging is the main clinical classification system for MB patients and evaluates the local extent (T stage) and the tumor’s dissemination (M stage) [31,32]. Although the role of the local extent as a prognostic factor is debated, with some studies showing worse prognosis for tumors located in the midline or with involvement of the fourth ventricle and the brainstem [26,33,34,35] and others not demonstrating similar findings [36,37], the contribution of metastasis to prognosis is well established. It is estimated that 30% of patients present with metastatic disease at diagnosis, which is accompanied by significantly diminished survival rates, with an eight-year OS of 65% and 27% in non-metastatic and metastatic patients, respectively [26]. Special consideration is given to stage M1 (tumor cells disseminated to CFS), which exhibits decreased OS similar to other metastatic stages, implying the need for prompt diagnosis of M1 staging, incorporation into high-risk features, and the implementation of intensifying treatment plans [24,26,38,39].

### 3.4. Extent of Resection

Surgical resection comprises an important part of MB treatment plans, and the extent of resection poses a significant prognostic factor. The main goal of resection is the removal of the whole extent of the tumor, with gross total resection (GTR) demonstrating a favorable impact on both PFS and OS, as shown by observational studies and meta-analyses [8,26,34,40]. On the other hand, residual disease after surgery, and especially residual tumors > 1.5 cm^2^, is considered a poor prognostic factor, leading to local tumor relapses and worst survival rates. However, the benefit of extensive surgical resections may be counterbalanced for post-surgical neurologically adverse events, ranging from endocrinologic abnormalities to neurologic complications such as cerebellar-mutism syndrome, which are present in a considerable percentage of patients after GTR [41]. The latter is of paramount importance mainly for centrally located tumors, in whom total resection might not be feasible and which are categorized as high-risk tumors with a need for treatment intensification. Despite that, Thompson et al., when examining the prognostic value of GTR compared to near-total resection (NTR), along with the integration of clinical and molecular factors, did not conclude significant survival differences, and similar results were also shown by other studies [42,43,44]. Consequently, GTR demonstrates a favorable impact on the prognosis of MB patients, but the EOR should be evaluated in combination with other parameters, such as tumor location and post-surgical complications.

### 3.5. Histological Variant

There are four main histological subtypes of MBs: desmoplastic/nodular (DMN), classic (CMB), MBs with extensive nodularity (MBEN), and large cell/anaplastic (LCA). Each of them is characterized by different histological patterns and is associated with distinct molecular and genetic alterations, exhibiting diverse prognosis [3,45,46]. Multiple studies have examined the role of histology as a prognostic factor of MBs. Firstly, DMN, which is characterized by desmoplasia with pericellular fibrinogen deposits, demonstrates better prognosis compared to the classic subtype ([8], desmoplastic vs. classic, HR 0.41, 95% CI 0.31–0.56, OS, and [26], DMN/MBEN vs. classic, HR 0.44, 95% CI 0.31–0.64). Similarly, MBEN histology, which is mainly found in the early years of life, exhibits and good to excellent prognosis [47]. The above survival advantage seems to be maintained even in the presence of adverse prognostic factors (i.e., metastatic setting). In a more detailed way, Leary et al. suggested a similar EFS in non-metastatic compared to metastatic DMN, whereas Gupta et al. showed a better OS in extracranial metastatic desmoplastic compared to non-desmoplastic subtypes [48,49]. On the other hand, LCA, which is characterized by the presence of anaplasia in histopathology, exhibits the worst prognosis compared to other subtypes [46]. Semantically, the degree of anaplasia seems to affect prognosis in a significant manner, where severe anaplasia leads to worse OS and EFS compared to mild. The poor prognosis of that subtype could be attributed to the association of LCA with high-risk features, as it usually affects patients at a younger age, is diagnosed with metastasis at presentation, and is associated with specific molecular and genetic alternations such as LOH, isochromosome 17q, and MYC-family genes [50,51,52]. Especially for the latter, Ellison et al. and Ryan et al. demonstrated the co-expression of c-MYC amplification with severe anaplasia and high-risk features, implying a worse prognosis [53,54].

Despite that, the latest WHO classification combines all the above histological subtypes into one category, called histologically defined MBs, which are associated with specific molecular pathways, suggesting the need for a multilayered evaluation of specific tumor characteristics.

### 3.6. Other Prognostic Factors

Except for the above-described clinicopathological and molecular factors, hematological and serum markers are also described in the literature, which can predict the survival of MB patients. Li et al. investigated the role of serum markers on prognosis and revealed that an elevated preoperative neutrophil-to-lymphocyte ratio (NLR) and platelet-to-lymphocyte ratio (PLR) were detected more frequently in Group 3 and Group 4 MBs and were associated independently with worse PFS and OS [55]. In addition, lower levels of lymphocytes during radiotherapy (RT) were associated with increased risk of recurrence [56]. In agreement with this, Zhu et al. evaluated the prognostic value of a systemic inflammatory index (SII) and nutritional status along with serum markers and advocated that high levels of inflammatory markers impaired OS in multivariable models [57].

Apart from serum markers, recent research has focused on the fast-growing field of radiomics, where the incorporation of image analysis into risk-prediction models, which are based on well-established factors, could evaluate the prognosis of each patient preoperatively and at diagnosis. The latter is of paramount importance, as it would assist with further understanding of the diverse nature of MBs and the designation of appropriate treatment strategies. Until recently, radiomic analysis had managed to indirectly predict the prognosis of patients, as MRI findings were associated with the molecular subgroup of the tumor [58,59] or the dissemination to the CSF [60]. However, the co-estimation of imaging findings, clinical characteristics, and molecular subgroup led to the development of nomograms, predicting both PFS [61] and OS [62].

## 4. Molecular Classification

During the last 20 years, knowledge about medulloblastoma biology has fiercely increased, primarily because of the advance of integrated genomics. The first approved classification of medulloblastomas concerning the molecular characteristics was performed by the WHO in 2016, resulting in four different molecular groups of MBs, each of which has a totally different molecular signature from both genetic and epigenetic aspects: WNT (wingless-related integration site) activated, SHH (sonic hedgehog)- activated, Group 3, and Group 4 [16,17,63]. The first two groups were identified and named based on the signaling pathways that were found to be activated in WNT MBs and SHH MBs, respectively: Wingless/Integration-1 (WNT) activated and sonic hedgehog (SHH) activated. It has been histologically proven that WNT MBs and SHH MBs arise from different cell types. It is well known that medulloblastomas are generated from cells that are related to some extent to cerebellar granule-neuron-precursor (CGNP) development and that some medulloblastoma cells retain primitive features equivalent to those of the precursors of the embryonic brain. Therefore, it seems that the acquisition of CGNP identity is a crucial determinant of progenitor cells’ ability to form hedgehog-induced MBs [64]. Group 3 and Group 4 constitute approximately 65% of medulloblastoma cases and are characterized by great heterogeneity in clinical phenotypes and survival rates [20]. These different subtypes are associated with different demographic and clinical features, but this is not always the case. Lately, a unified lineage of origin for both Groups 3 and 4 within the human fetal RL^SVZ^ was defined. These recent data explain the underlying molecular signatures, biological and clinical overlap, and site of diagnosis that these two groups share [5].

The intention behind molecular classification is to better relate clinical phenotypes with the tumor biology and to identify new personalized, safer, therapeutic targets. The four main molecular groups describe in a vague manner the disease evolution and outcome. In the updated WHO classification of the CNS tumors, Group 3 and Group 4 are merged into one, called non-WNT/non-SHH MB. It is a very large category, to which the majority of pediatric MB patients belong [21]. The classification by Schwalbe et al. divides MBs into seven subtypes and impresses that the only group that remains intact is the WNT-MB class.

### 4.1. The WNT Group

The WNT group accounts for 10% of MBs and usually develops in the midline cerebellum but is also capable of spreading to the dorsal brainstem. The WNT group overall has the best prognosis out of all MB groups [65]. Mutations of the WNT-signaling pathway cause its fundamental activation [66]. Genomic analyses showed that the vast majority of patients (85–90%) with WNT MBs have a mutation in exon 3 of CTNNB1 [10]. As a result, β-catenin is stabilized, leading to constant activation of the WNT pathway [67]. Mutation in adenomatous polyposis coli (APC), a germline mutation, is also associated with WNT MBs [62].

There is a conflict about the subtypes within the WNT group; some studies have presented evidence for at least two distinct subgroups, whereas other researchers have reported only one [68,69,70]. One of the suggested subgroups is low risk, more than 90% survival, and typically non-metastatic [10]. The second suggested WNT subtype is mainly characterized by metastatic cases and anaplastic histology [68]. Because of the confusion concerning WNT-MB sub-classification, many subdivisions have been proposed. Some groups use both testing of gene-expression patterns with DNA-methylation arrays. A very recent and modern approach is the similarity-network-fusion (SFN) strategy, which constructs networks of combined data [70]. As a result, Cavalli et al. identified two different subtypes of WNT MBs: WNTα and WNTβ. Generally, WNT MBs are related to accumulated beta-catenin inside the nucleus, accumulation of mutations in CTNNB1, and, in most cases, monosomy in chromosome 6 [66]. The above-mentioned markers are used to identify WNT MBs, but 1 in 10 MBs can be missed [71].

### 4.2. The SHH-MB Group

SHH MBs are the most common group in infants, accounting for 25% of all medulloblastoma cases. Usually, it is found in cerebellar hemispheres but can also be found in the midline. It is characterized by mutations or copy-number alterations of SHH-pathway genes. According to Robinson et al., the infant-SHH group should be split into SHH-I and SHH-II, as it seems that the first one is enriched in SUFU aberrations and chromosome 2 gain [72,73,74]. Another study suggested the subclassification of the SHH group into α, β, γ, and δ subgroups [66,70]. According to the study, infant SHH-I and SHH-II correspond to SHH-β and SHH-γ, respectively. The prognosis of SHH-γ is better than that of SHH-β. SHHα can be of the LCA or ND subtype. It has been found to be enriched with MYCN, GLI2, and YAP1 amplifications, as well as TP53 mutations and copy-number alterations (9q, 10q, and 17p loss) [70,75,76].

### 4.3. Group 3 and Group 4

Group 3 has the highest rate of mortality, with only a 58% 5-year OS in children and an even lower, 45% 5-year OS in infants [19,65,77]. In fact, Group 3 has a variety of grim features, such as young age and metastasis even after diagnosis. Histologically, it is characterized by anaplastic large cells that can serve as a prognostic marker [78]. Concerning molecular events, an aberrant amplification of MYC takes place [79]. Group 3 seems to be highly heterogenous with patients developing MYC-amplified tumors, with a very short survival rate since only 20% survive up to 5 years. Group 3 MB tumors reappear as metastases far from the primary tumor, with a rate of metastases independent of the survival percentage. Tumors belonging to Group 4 usually demonstrate isochromosome 17q (i17q) [80]. The overlap in the mutational spectrum, the transcriptional profile, and the DNA-methylation pattern between Group 3 and Group 4 has led them to be considered the non-WNT/non-SHH group in the latest WHO classification [21]. Indeed, tumors belonging to this group have more of a molecular affinity with each other than with SHH or WNT MBs. Unlike WNT and SHH MBs, the reason for this molecular diversity and the large and confusing overlapping between Groups 3 and 4 remains obscure.

In summary, since the recognition of the four core MB groups in 2010, molecular subgrouping has experienced a revolutionary diversification. Nowadays, about 12 molecular subtypes are defined and new data come to light every day. Cavalli et al. analyzed more than 700 primary-MB tissue samples and showed that SHH MBs can be divided into four subclasses, with different copy-number aberrations, activated pathways, and—most importantly—different clinical outcomes. The SHHα subgroup has the worst prognosis of all four subgroups [70].

## 5. Medulloblastomas and Genetics

Medulloblastoma genetic analysis is a key component not only for risk stratification but also for prognosis [81]. Currently, the molecular classification of MB tumors is routinely performed not only because of the impact that variations have on treatment and clinical outcome but also because it is undeniable that the great progress in molecular analyses, such as high-throughput next-generation-sequencing (NGS) technologies, allows for better understanding of medulloblastomas’ tumorigenesis and biology, which should ultimately enhance the application of tailored treatment therapies.

Each molecular subgroup of MBs can be identified by a unique signature of DNA alterations and copy-number variation, abnormal gene transcription, and various post-transcriptional modifications. These characteristics are associated with different clinical phenotypes and, when combined, could have an important prognostic value. TP53 R273H mutation, which lies within the DNA-binding domain of the tumor-suppressor protein Tp53, is responsible for the loss of DNA binding and decreased activation of Tp53 target gene expression. As a result, cells resist apoptosis and an aberrant transcriptional activation and increased cell migration take place. TP53 mutation in the R273H point is associated with poor follow-up in the SHHα subgroup and generally with worse prognosis [70]. In addition, it has been shown in mice models that TP53 R237H mutation is responsible for tumors with high metastatic potential [82,83]. TP53 germ-line mutations lead to mutations in hedgehog-signaling genes, resulting in medulloblastoma growth in patients with Li–Fraumeni syndrome. Cerebellum granule-cell precursors (GCPs) are the cells of origin of SHH medulloblastomas, indicating that GCPs are highly susceptible to tumorigenesis in the absence of P53.

Other genes, such as MYCN and GLI2, are frequently found in various cohorts to be over-expressed [84]. Indeed, both genes are risk factors for SHH MBs. TP53 could serve as a reliable prognostic marker in SHH MB cases, as it has been recently shown that patients with the above-mentioned mutations have a worse outcome compared with patients with wild-type TP53 [85]. From a histological perspective, mutations in the TP53, when present, reflect poor prognosis in all four groups [86]. A validated molecular marker used for Group 3 is NPR3 (natriuretic peptide receptor 3), a protein-coding gene, and for Group 4 it is KCNA1 (potassium voltage-gated channel subfamily A member 1), another protein-coding gene [66]. Apart from the conventional genetic markers, it has been demonstrated that the incorporation of DNA-methylation biomarkers significantly ameliorates survival prediction for non-WNT medulloblastomas in patients 3 to 16 years old. The addition of MXI1- and IL8-methylation status with the currently used molecular and clinical features noticeably improved disease-risk stratification [87,88].

It may take years before genetic tests based on sequencing technologies can be incorporated as a part of standard care for our patients. Further studies are needed to validate their prognostic merit and lead to tailored therapies that are able to detect measurable, residual disease and prevent the fatal relapse of medulloblastomas.

## 6. Non-Coding RNAs

New reliable and affordable methods are needed to help prognosis and to suggest the most appropriate therapy to patients with MBs individually. MiRNAs could be the answer to that, as they have already been used in treating various diseases, including cancer. MicroRNAs (miRNAs or miRs) are small non-coding RNA molecules that have been identified as major regulators of cancer growth, functioning either as oncogenes or tumor suppressors. MiRNAs help tumor cells called oncomiRs to proliferate, but others inhibit cell proliferation and promote cell differentiation, known as tumor-suppressor miRs. MicroRNAs have been implicated in gene-expression regulation through different mechanisms. Usually, based on sequence-specific binding, they can inhibit the translation of messenger RNA (mRNA) or mark mRNA molecules for degradation [89]. Due to molecular-technique improvement, new and affordable methods are available, suggesting that miRNAs can be used as promising biomarkers of MBs [90,91]. MiRNAs can be readily detected in tumor biopsies, and they are also stable in body fluids due to being bound to lipoproteins, associated with the Argonaute 2 (Ago2) protein, packaged into microvescicles and other microparticles (such as apoptotic bodies, microvesicles, and exosome-like particles) as circulating miRNAs [92,93,94]. Therefore, they are protected from endogenous RNA activity, making them a reliable and stable marker [95].

Different miRNAs have different molecular signatures in the four distinct groups. It has also been found that downregulation of miR-204 expression is associated with poor survival in children diagnosed with Group 3 or Group 4 MBs [96]. Moreover, in Group 4 patients, there is generally an intermediate prognosis: Low expression of miR-204 marks out a distinct sub-group with remarkably poor survival. This is a rather important finding, as Group 4 is characterized by a lack of reliable prognostic markers [97].

The most important remark about miR-204 is probably that the replacement of downregulated expression levels could limit a tumor’s metastatic potential and thus be a promising therapeutic approach. The improvement of RNA-sequencing platforms has led to the identification of several miRNAs that alter gene regulation and promote cancer and metastasis. Large cohort studies with the neuroblastoma pediatric population revealed the crucial role of miR-383, miR-206, miR-183, miR-128a/b, and miR-133b in WNT MBs, as they are downregulated significantly [98,99]. Identification of new molecular markers for diagnosis and prognosis is important. Long non-coding RNAs (lncRNAs) are now among the most promising diagnostic tool, as they play an important role in brain-tumor growth and metastasis [100]. Kesherwani et al. found that high expression of DLEU2 and DSCR8 in Group 3 and high expression of DLEU2 and low expression of XIST in Group 4 are associated with poor prognosis of MBs [101]. Gao et al. showed that LOXL1-AS1 (LOXL1 antisense RNA 1) promotes cell proliferation and migration in MBs [102,103,104]. In 2020, Joshi et al. conducted the first genome-wide analysis of lncRNAs’ expression profile in MBs. The screening of 17 lncRNAs yielded positive results, with H19, lnc-RRM2–3, LINC01551, LINC00336, lnc-CDYL-1, FAM222A-AS1, and AL139393.2 associated with poor prognosis [105].

In 2022, Lee et al. showed in vitro that long non-coding RNA SPRIGHTLY promote the MB subgroup 4 [106]. SPRIGHTLY modulates the EGFR-signaling pathway through regulatory effects. These modifications affect cell growth through changes in the tumor microenvironment [106].

According to Zhang et al., HOTAIR (HOX transcript antisense RNA) is another LncRNA that promotes MB progression [104]. HOTAIR knockdown led to a reduction of tumor growth, cell proliferation, and increased cell apoptosis. HOTAIR act through bindings to miR-1 and miR-206, which lead to increased expression of YY1 (Yin Yang 1). It has been proven that non-coding RNAs regulate tumor metabolism involved in development and relapse. We estimate that future studies will enable a full investigation into ncRNA-associated prognostic potential and that it will be incorporated into the prognosis and stratification of medulloblastomas.

## 7. Recurrence

Recurrence of MB (relapsed MB, rMB) is considered a serious adverse event of the tumor, which is present in approximately 30% of patients and associated with poor prognosis. Recent studies have reported significantly diminished PFS and OS for rMBs (only 5% of patients will remain alive after 5 years), even in the presence of a wide range of therapeutic options, such as re-irradiation, surgery, chemotherapy, and targeted therapies [107]. The above observation prioritizes the need for the development of appropriate risk-stratification models based on clinical and molecular subgroups for relapsed patients, which could also assist with the selection of adequate treatment strategies.

Regarding prognostic factors of MB relapse, both clinical and histological characteristics of the tumor at diagnosis seem to affect the time and pattern of relapse and post-relapse survival rates. Concerning age, younger patients (<5 years old) have a higher rate of recurrence, and the role of histology is not clear. Although LCA was associated with reduced time to relapse and short time to death post-relapse [108], Sabel et al. argued that histology did not affect the aforementioned factors [109]. In addition, the detection of MYC amplification was characterized as a negative prognostic for both the development of recurrence and time to death after relapse [109]. Treatment modality at diagnosis was another significant factor in observational studies, where craniospinal irradiation of the primary tumor showed prolonged time to relapse and better survival rates afterwards [108,109,110]. Nevertheless, the site of recurrence seems not to be affected by treatment type and is mainly molecularly driven. On the other hand, presence of metastatic dissemination at diagnosis had no effect on survival after relapse [111].

Prospective studies also evaluated the existence of prognostic factors for MB recurrence in infants (iMB) and young children and investigated parameters associated with post-relapse survival (PRS). Gross total resection and the absence of residual disease or <1.5 cm^2^ residual disease showed a better EFS compared to STR or residual > 1.5 cm^2^ [112,113], whereas the time from diagnosis to relapse was not shown to affect survival [114]. From a histopathological perspective, DMEN/DN histology demonstrated a better PFS compared to CMB [112], and further molecular analysis revealed that a considerable number of patients within the SHH subgroup had DN/MBEN histology and were associated with better prognosis. However, Hicks et al. argued that in DN/MBEN SHH iMBs, there is a subset of patients with the SHH_I_ molecular subtype that had a worse PFS (HR 3.8, 95% CI 1.00–11.8, *p* = 0.038), underlining the importance of an extended molecular subtyping for the better understanding of recurrence risk in iMBs [115]. In concordance with this, the SJYC07 trial reported similar results [72], whereas the HIT-2000 study [116] and ACNS1221 [117] did not reach statistical significance for the difference in PFS between the SHH_I_ and SHH_II_ subgroups. On the contrary, non-DN/MBEN histology or STR within the SHH subgroup were considered poor prognostic factors. On the other hand, Group 3 usually presented with a disseminated relapse pattern [115,118] and was characterized by MYC amplification and LCA histology, resulting in diminished PFS, whereas Group 4 demonstrated local relapse and a moderate prognosis. Regarding PRS and taking under consideration that CSI is often omitted in iMBs due to neurocognitive disorders, salvaging CSI led to improved survival after recurrence [114,118]. Other prognostic factors for PRS, as shown by multivariable models, were Group 3 and a disseminated pattern of relapse, which led to poor prognosis.

The observation of different treatment outcomes among MB patients, their diversity in histopathological and molecular characteristics, and the presence of different cells of origin, imply the detection of discrete molecular findings at relapse compared to the primary tumor. However, methylation data analysis performed at relapsed tumor specimens concluded that MBs maintain their primary characteristics also at recurrence (“tumor fidelity”), affecting all aspects of the disease, starting from time to relapse [119]. Differences in relapsed MBs are critical to the development of effective therapies. Research on primary and recurrent MB tumors has revealed that the variance in genomic changes between the two is the mutations on the driver that are present in around 41% of reoccurring tumors and various acquired somatic DNA alterations in around 53% of them [120,121]. The performance of methylation profiling indicates, once more, that in general, the molecular signature of the relapsed tumor resembles that of the original one, except, of course, for the prementioned alterations [122].

In the SHH MB group, it seems that age is being negatively correlated with differences between gene expression in the primary and in the relapsed tumor. Particularly, the youngest patients with SHH MB, develop relapsed tumors with copy-number variations and supreme transcriptome variability compared to the primary tumor. The majority of relapsed cases are characterized by the loss of 17p and, in a limited number of cases, by new TP53 mutation [123]. This finding suggests a model of bi-allelic gene inactivation, resulting in further accumulation of genomic changes. On the contrary, Groups 3 and 4 are presented with metastatic disease, which is associated with poor prognosis [124].

Richardson et al. studied more than 100 relapsed MBs, enabling the characterization of the molecular basis in medulloblastoma reoccurrence with the goal of the clinical exploitation of this knowledge. They found that molecular groups and the novel subtypes also remained consistent to the original stratification of the primary tumor. However, it was found that a small subset of Group 4 MBs switched subgroups when relapsing [125].

Hill et al. also proved that MBs show an altered molecular profile at relapse, which is predictive of disease prognosis but cannot be detected earlier. They pointed out the significance of P53-MYC interactions during tumor relapse and their prognostic value as predictors of clinically aggressive disease [126]. These findings enhance the need for biopsy in clinical practice during relapse and thus the development of improved and patient-tailored therapeutic strategies.

## 8. Applying the Knowledge Gained

The knowledge acquired from molecular data suggests that clinical risk is not enough for the reliable assessment of relapse risk. The intention behind molecular classification is to better relate clinical phenotypes with the tumor biology and to identify new personalized, safer, therapeutic targets. So far, randomized controlled trials focusing on older children, and SJYC07 including younger children (<3 years old), have evaluated both clinical prognostic factors and molecular subgroups to guide treatment decisions. More precisely, the ACNS0331 study concluded a non-inferior EFS in clinically defined average-risk patients with MB in whom a reduction of radiation-boost volume was preferred compared to posterior-fossa radiation therapy. In addition, that regimen decreased neurocognitive disorders attributed to RT, serving as an additional advantage in the treatment of patients lacking high-risk features. However, molecular subgrouping revealed a specific subgroup with inferior EFS when low-dose CSI was administered compared to the standard dose [127]. In concordance, the SJMB3 trial stratified patients into categories based on both clinical and molecular prognostic factors, pinpointing the importance of co-estimation of those parameters in the case of treatment de-intensification in favorable-risk groups (such as the WNT subgroup) or intensifying treatment in the presence of adverse prognostic factors such as metastatic disease or subtype III or MYC amplification in Group 3 patients [78]. Similarly for younger children enrolled in the SJYC07 trial, treatment selection according to clinically and histologically defined risk-adapted factors did not improve EFS, especially for the intermediate- and high-risk groups [78]. On the contrary, using molecular profiling led to a better survival outcome in the SHH_II_ subgroup compared to SHH_I_, in whom significant adverse genetic factors, such as SUFU aberration and isochromosome 2 gain, were detected. In addition, recent clinical trials have been designed (i.e., PNET 5 (NCT02066220) and SJMB12 (NCT01878617)) and enrolled pediatric patients based on both clinical and molecular data.

For the PNET 5 study, the only criterion for the assignment of patients was β-catenin status. Therefore, children positive for β-catenin endonucleic accumulation were assigned to treatment arm PNET5 MB-LR, whereas children with negative results were assigned to treatment arm PNET 5 MB-SR. It is worth mentioning that the initial diagnostic assessments (imaging, staging, histology, and tumor biology) needed for inclusion in the study were the same for both treatment arms. An amendment of the protocol permitted the entry of WNT-activated MB patients with high-risk characteristics to the PNET 5 MB WNT-HR study. Likewise, patients with high-risk SHH MB with TP53 mutation (somatic, germline, and mosaicism) were included in the PNET 5 MB SHH-TP53 study.

The second phase II clinical trial, called SJMB12, stratified MB treatment according to clinical risk (low, standard, intermediate, or high risk) and molecular subtype (WNT, SHH, or non-WNT/non-SHH). The main aim was to evaluate whether participants with low-risk WNT tumors could be treated with a lower dose of radiation and mild chemotherapy to achieve the same survival rates with fewer side effects. All children had surgery to remove as much of the primary tumor as was safely possible, radiation therapy, and chemotherapy. However, the amount of therapy (radiation and chemotherapy) was determined by the patient’s treatment stratum. The treatment-stratum assignment had been planned based on the tumor’s molecular subgroup and clinical risk.

Both studies are ongoing, and we have to wait for the results to fulfill the research community’s ambitions for more efficient and safe treatment of MBs.

## 9. Conclusions

Until 2014, MB grading, like general grading in CNS tumors, was based exclusively on histological data. However, since then, a lot has changed in the diagnostics and therapeutics of cancer diseases. Undoubtedly, genomic and transcriptomic analyses shed light on the molecular and genetic pathways involved in the pathogenesis of MBs, evolving risk-stratification systems. Molecular markers can provide powerful prognostic knowledge, and this is why they have been added to the biomarker list of grading and prognosis estimation. Apart from the already-applied technologies, new molecular biomarkers have come into view. Recent studies have revealed the promising prognostic value of various analyses based on genomic approaches that use the cell-free DNA (cfDNA) of the cerebrospinal fluid (CSF) and the different structural variants and point mutations by multiplexed droplet digital PCR (ddPCR) [128]. Moreover, Cao et al. brought out the great potential of the MCM3 marker, a minichromosome-maintenance protein, through a multi-omics approach that combines single-cell RNA sequencing and proteomics analysis [129]. Undoubtedly, scientists are trying to combine state-of-the-art molecular analyses with modern mass-spectrometry imaging techniques that will allow for the identification of sub-group-specific tumor-cell populations whose unique functions can be exploited for future prognostic and therapeutic strategies.

Disparities among patients’ clinical outcomes make the application of further subtypes of MB urgent and have been included in this study. Despite the prognostic value of the recent 2021 WHO molecular classification, it is obvious that there is a great heterogeneity within each of the four groups and their relevant subgroups. Therefore, there is a constant update of downstream classification from studies around the world, and this is only the beginning. As technology improves and permits wider and more complicated data interpretation, a more detailed subgroup analysis will appear. Furthermore, as the new molecular characterization in MBs remains independent and difficult to match with the histological data, we face problems of overlapping associations. That is the case for desmoplastic/nodular MB and MBEN, subtypes of the SHH group [130].

The new era in molecular-based medulloblastoma prognosis and stratification has arrived. It remains to be seen, while awaiting the first sets of data from the ongoing relevant clinical trials, how translational research can be integrated into clinical practice to balance survival with quality of survival.
